# The Neuroprotective Role of Ginsenoside Rg1 Against Cerebral Ischemia–Reperfusion Damage Through Inhibition of Mitophagy via Blocking Mitophagosome‐Lysosome Fusion

**DOI:** 10.1111/cpr.70071

**Published:** 2025-06-03

**Authors:** Lin Ai, Hangui Ren, Yuan Wang, Mengfan Liu, Yufei Qiu, Jiling Feng, Rongchen Dai, Wang Fu, Yongpeng Wang, Zhichao Xi, Hongxi Xu, Feng Wang

**Affiliations:** ^1^ Department of Neurology Seventh People's Hospital of Shanghai University of Traditional Chinese Medicine Shanghai China; ^2^ School of Pharmacy Shanghai University of Traditional Chinese Medicine Shanghai China; ^3^ Engineering Research Center of Shanghai Colleges for TCM New Drug Discovery Shanghai China; ^4^ Yueyang Hospital of Integrated Traditional Chinese and Western Medicine Shanghai University of Traditional Chinese Medicine Shanghai China; ^5^ Shanghai General Hospital Shanghai Jiao Tong University School of Medicine Shanghai China

**Keywords:** cerebral ischemia–reperfusion injury, ginsenoside Rg1, ischemic stroke, mitophagosome‐lysosome fusion, mitophagy

## Abstract

Ginsenoside Rg1 has shown promise in ameliorating cerebral ischemia–reperfusion injury (CIRI). However, its precise molecular mechanisms remain unclear. In this study, an in vitro CIRI model was established using SH‐SY5Y and SK‐N‐AS neuronal cell lines subjected to oxygen–glucose deprivation followed by reoxygenation (OGD/R). For the in vivo model, C57BL/6J mice underwent middle cerebral artery occlusion and subsequent reperfusion (MCAO/R). The protective effects of Rg1 against OGD/R injury were analysed using the CCK‐8 assay and the PI exclusion method. The in vivo neuroprotective effects of Rg1 against CIRI were evaluated using various assessments, including brain blood flow, neurological deficits, behavioural tests, TTC, H&E, Nissl and TUNEL staining. Mitophagy was assessed by detecting mitophagy‐initiating proteins via Western blotting, transmission electron microscopy, immunohistochemistry and immunofluorescence staining. Additionally, mitochondrial function was assessed by ATP measurement, the JC‐1 assay and MitoSOX‐based flow cytometry. Our results show that Rg1 significantly mitigated cell death caused by OGD/R and substantially enhanced cell viability in vitro. Moreover, Rg1 alleviated OGD/R‐induced mitochondrial dysfunction, as indicated by preserved mitochondrial membrane potential and decreased mitochondrial ROS levels. Mitophagy was induced after OGD treatment, which was subsequently inhibited by Rg1 during reperfusion. Mechanistically, Rg1 disrupted the fusion of mitophagosomes with lysosomes rather than inhibiting mitophagy initiation, leading to an accumulation of mitochondrial proteins and mitophagy‐initiating proteins. Notably, prolonged inhibition of mitophagy by Rg1 did not induce cytotoxicity or exacerbate mitochondrial dysfunction. Furthermore, administration of Rg1 in MCAO/R mice significantly improved brain blood reperfusion, reduced infarct volume, improved neurological deficits, preserved brain tissue integrity and decreased neuronal apoptosis. Consistent with the in vitro observations, Rg1 upregulated mitophagy‐related protein expression in MCAO/R mouse brain tissues, indicating potential inhibition of mitophagy. In conclusion, our study reveals that Rg1 significantly alleviates CIRI at least partially by suppressing mitophagy, specifically by impeding the fusion of mitophagosomes with lysosomes.

## Graphical abstract

The present study revealed the neuroprotective effect of ginsenoside Rg1 against cerebral ischemia–reperfusion injury through inhibition of mitophagy via blocking mitophagosome‐lysosome fusion both in vivo and in vitro models.

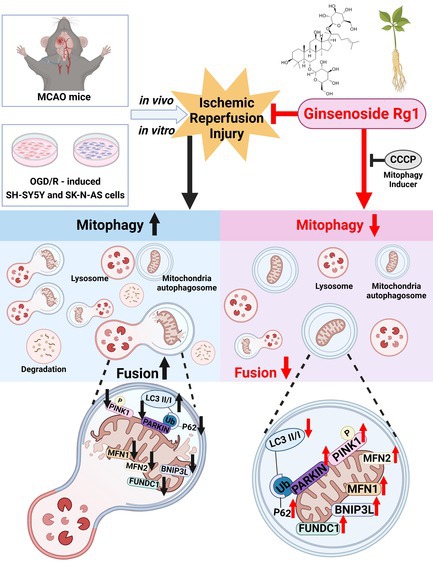

## 1 Introduction

Cerebral ischemia–reperfusion injury (CIRI), characterised by a transient interruption and subsequent reestablishment of cerebral blood flow, poses a significant clinical challenge due to its devastating consequences, including neuronal cell death, neurological deficits and cognitive impairment [1]. However, existing treatments for CIRI are lacking, and effective drugs need to be further explored. Ginsenoside Rg1 (Rg1), a bioactive compound derived from the roots of *Panax ginseng* C. A. Meyer, is highly regarded in traditional medicine for its broad therapeutic potential. It is beneficial in treating various pathological conditions, including cardiovascular disorders [2], neurodegenerative ailments [3] and CIRI [4]. Rg1 has been demonstrated to be beneficial for neuroprotection and improvement of neurological outcomes post‐ischemic stroke through pleiotropic effects, including anti‐inflammatory [5], antioxidant [6] and anti‐pyroptotic actions [7]. Notably, our previous study found that Rg1 induces mTOR‐regulated lysosomal dysfunction, thereby inhibiting autophagy and preventing CIRI, underscoring the crucial role of autophagy in CIRI protection [8]. However, despite these promising findings, the precise mechanisms through which Rg1 exerts its therapeutic effects in CIRI remain incompletely understood.

Mitochondria are essential for neuronal homeostasis, and their dysfunction can trigger a cascade of cellular events, including apoptosis and necrosis, inflammation and the generation of reactive oxygen species (ROS), all of which are associated with CIRI [9, 10, 11]. Mitophagy, a specialised form of autophagy, is a critical cellular process that eliminates impaired or dysfunctional mitochondria, thereby preserving mitochondrial mass and maintaining cellular homeostasis [12]. This process is initiated by PTEN‐induced putative kinase 1 (PINK1), which phosphorylates ubiquitin, thereby triggering the activation of E3 ubiquitin ligase Parkin. Parkin then facilitates the polyubiquitination of mitochondrial outer membrane proteins, promoting the recruitment of mitophagy receptors. Additionally, mitophagy can also be initiated independently of ubiquitin by BCL2 and adenovirus E1B 19 kDa‐interacting protein 3 (BNIP3) and its counterpart BNIP3‐like (BNIP3L), which contribute to the removal of impaired mitochondria and the preservation of cellular function [13]. The final stage of mitophagy involves the engulfment of damaged mitochondria within autophagosomes, forming mitophagosomes that then fuse with lysosomes. This fusion results in the degradation of mitochondrial components, which are recycled into fundamental building blocks such as amino acids, lipids, and carbohydrates [14]. The process of mitophagosome biogenesis is crucial, as an accumulation of mitophagosomes can impede the induction of mitophagy [15]. Notably, the formation of mitophagosomes and the sequestration of mitochondria can occur even in the absence of Parkin [16]. Following cerebral ischemia, the reperfusion phase is pivotal, as it can shift mitophagy from being a protective to a detrimental role [17]. Therefore, regulating mitophagy, particularly mitophagosome biogenesis, could be a promising strategy for alleviating CIRI.

In this study, we observed a significant reduction in CIRI following Rg1 treatment in both OGD/R and MCAO/R models. These findings suggest that Rg1's neuroprotective effects may be at least partially due its ability to suppress excessive mitophagy. Mechanistically, Rg1 hinders the fusion of mitophagosomes with lysosomes rather than inhibiting the initiation of mitophagy, thereby preventing mitochondrial degradation. In conclusion, Rg1 demonstrates potential as a therapeutic candidate for mitigating cerebral CIRI. Further research is needed to fully elucidate its mechanisms of action and to explore its potential clinical applications in the treatment of CIRI.

## 2 Methods and Materials

### 2.1 Drugs and Reagents

Ginsenoside Rg1 (98% purity, 22,427‐39‐0) was obtained from AlfaBiotech Co. (Chengdu, China), dissolved in DMSO, and kept at −20°C for storage. We used the following primary antibodies in our study: PINK1 (23274‐1‐AP, 1:1000), MFN1 (13798‐1‐AP, 1:1000), MFN2 (12186‐1‐AP, 1:1000), SQSTM1/P62 (18420‐1‐AP, 1:1000), and GAPDH (60004‐1‐Ig, 1:10000) from Proteintech (Hubei, China); BNIP3L (A6283, 1:1000) and FUNDC1 (A16318, 1:1000) from ABclonal (Hubei, China); Parkin (2132, 1:1000) from Cell Signalling Technology (MA, USA); and LC3 (L7543, 1:1000) from Sigma‐Aldrich (MO, USA). The secondary antibodies HRP‐Goat Anti‐mouse (5450‐0011, 1:2500) and HRP‐Goat Anti‐rabbit (5450‐0010, 1:2500) were used with the respective primary antibodies and sourced from Kirkegaard & Perry Laboratories (MD, USA). Other reagents included Mdivi‐1 (T1907) and cell counting kit‐8 (CCK‐8) from Topscience (Shanghai, China); CCCP (C2006‐4) and MitoTracker (C1048) and JC‐1 staining assay kit (C2006‐3) from Beyotime (Shanghai, China); and LysoTracker (L7528) from Life Technologies (CA, USA). MitoSOX Red (40778ES50, 1:2000) and ATP measurement kit (40210ES10) were purchased from YEASN (Shanghai, China). DAPI (G1012) and NeuN (GB15138) were obtained from Servicebio Technology Co. (Hubei, China).

### 2.2 Cell Culture and OGD/R Model

The SH‐SY5Y and SK‐N‐AS cell lines were cultured at 37°C in a humidified incubator with 5% CO_2_, following the protocols described previously [8]. The cells were grown in Dulbecco's Modified Eagle's Medium (DMEM, Gibco 6,123,113, MA, USA) supplemented with 10% foetal bovine serum and 1% penicillin–streptomycin. An OGD/R model was set up following the protocols described previously to simulate CIRI [8]. In brief, after the cells were plated and grown for 24 h, they were washed with PBS and then exposed to hypoxic conditions provided by Mitsubishi Gas Chemical's AnaeroPack Aero (Tokyo, Japan) in an environment containing glucose‐free DMEM supplied by MeilunBio (MA0851, Liaoning, China). The SK‐N‐AS cells received 12 h hypoxic treatment, while SH‐SY5Y cells received 10 h. Following the hypoxic phase, the cells were transitioned to simulate reperfusion by restoring DMEM and returning to normal oxygen conditions for an additional 24 h. Upon the initiation of reperfusion, various treatments were introduced to the cell cultures. These included DMSO, Rg1, Mdivi‐1 and CCCP, which were applied to the cells and maintained for 24 h.

### 2.3 Evaluation of Cell Viability by CCK‐8 Assay

SH‐SY5Y and SK‐N‐AS cells were cultivated in 96‐well plates at a concentration of 10,000 cells per well and were subjected to OGD/R conditions for 24 h. Following a 24‐h exposure to different concentrations of Rg1, CCCP, or a mixture of both, 10 μL of CCK‐8 solution was added to each well. After 3‐h incubation at 37°C, absorbance was measured at 450 nm using the EnSpire Multimode Plate Reader from PerkinElmer, USA.

### 2.4 Propidium Iodide (PI) Exclusion

The cell death ratio was evaluated using the PI exclusion assay [18]. Briefly, cells were harvested 24 h after administration of various drugs and then incubated with PI for 15 min. PI exclusion analysis was conducted using a flow cytometer Beckman Coulter (CA, USA).

### 2.5 Immunofluorescence Staining

The immunofluorescence staining procedure has been previously described [8]. For organelle co‐localisation, cells were stained with MitoTracker (1:5000) and LysoTracker (1:5000) for 1 h. Cells were then fixed with 4% paraformaldehyde (PFA) for 10 min and incubated with DAPI (Southern Biotech, AL, USA) overnight. To examine co‐localisation of mitophagy initiation proteins with lysosomes, SK‐N‐AS cells were stained with LysoTracker, fixed with 4% paraformaldehyde, and blocked with a 1% BSA solution for 60 min. Subsequently, the cells were exposed to primary antibodies against PINK1, Parkin, BNIP3L or FUNDC1 at 4°C overnight. After washing with PBS, cells were treated with Alexa Fluor 594‐conjugated secondary antibodies for 60 min at room temperature. The nuclei were visualised with DAPI, and the images were visualised by a fluorescence microscopy. The procedure was performed in triplicate, and co‐localisation was quantified using ImageJ software, analysing six distinct fields of view per replicate. To evaluate neuron‐specific mitophagy, brain sections were processed for double immunofluorescence staining. Briefly, frozen coronal sections were fixed, permeabilised, and incubated overnight at 4°C with primary antibodies against NeuN (1:500, neuronal marker, red) and MFN1 (1:300, mitophagy marker, green). The sections were then incubated with fluorophore‐conjugated secondary antibodies and counterstained with DAPI (blue) for nuclear visualisation. Images were acquired using a fluorescence microscope, and co‐localisation analysis was performed with ImageJ.

### 2.6 Transmission Electron Microscopy

SK‐N‐AS cells were cultivated in 10 cm dishes and either left untreated or treated with Rg1 for 24 h under OGD/R conditions. After treatment, the cells were dehydrated, infiltrated and embedded in SPON12 resin. The embedded samples were polymerised at 60°C for 48 h. Ultrathin sections (70 nm) were cut with a microtome, collected on copper grids coated with a Formvar film (mesh size 100). The sections were subsequently stained with uranyl acetate and lead citrate. After staining, the sections were air‐dried. The samples were then examined using a high‐resolution Talos L120C G2 transmission electron microscope (ThermoScientific, MA, USA), which enabled detailed visualisation of cellular structures at the nanoscale and provided insights into the effects of Rg1 treatment.

### 2.7 ATP Measurement

Intracellular ATP levels were quantified using the ATP luminescent cell viability assay kit. SH‐SY5Y and SK‐N‐AS cells (10,000 cells/well in 96‐well plates) underwent OGD/R, followed by 24‐h treatment with various concentrations of Rg1. After adding 100 μL of luciferase reagent, luminescence was measured at 560 nm using the EnSpire Multimode Plate Reader (PerkinElmer, USA).

### 2.8 Mitochondrial Reactive Oxygen Species (mtROS) Detection

Mitochondrial superoxide production was assessed by detecting mtROS levels using MitoSOX Red, a mitochondrial superoxide indicator. SK‐N‐AS and SH‐SY5Y cells were plated and subjected to OGD/R, followed by 24 h of treatment with Rg1. After incubation with MitoSOX Red at 37°C for 20 min, the cells were washed with PBS. Fluorescence intensity was then quantified using a flow cytometer, and data was analysed with FlowJo software.

### 2.9 Mitochondrial Membrane Potential (MMP) Detection

The MMP was evaluated using the JC‐1 staining kit according to the manufacturer's instructions. SK‐N‐AS and SH‐SY5Y cells were treated as described above. After 20 min of incubation with JC‐1 at 37°C, the cells were centrifuged at 600 g for 3 min and washed twice with PBS. Flow cytometry was used to analyse the cells. JC‐1 aggregates exhibit red fluorescence, while its monomeric form in the cytoplasm emits green fluorescence. An increase in green fluorescence intensity indicates mitochondrial depolarisation.

### 2.10 Western Blotting Analysis

The Western blotting procedure was performed as described previously [19]. After treatment, cellular lysates were prepared on ice using a RIPA buffer with protease/phosphatase inhibitor cocktails. The protein content was quantified using a BCA protein assay kit (Meilunbio, Dalian, China). Proteins from each sample were denatured, separated by SDS‐PAGE, and transferred to PVDF membranes. The membranes were blocked with 10% non‐fat milk for 90 min, then incubated with primary and secondary antibodies. The bands were detected and recorded using an ImageQuant LAS 4000 system.

### 2.11 Animals

Male C57BL/6J mice (8 weeks of age) were sourced from Beijing Charles River Laboratories, China. All animal experimental procedures adhered to established ethical standards and were approved by the Ethics Committee of Shanghai University of Traditional Chinese Medicine (Approval number: PZSHUTCM2402240002).

### 2.12 Establishment of Transient Middle Cerebral Artery Occlusion/Reperfusion (MCAO/R) Mouse Model

Mice (22 to 25 g) were subjected to the MCAO/R model as described previously [8, 20]. Mice were anaesthetised with isoflurane, and the body temperatures were monitored and maintained within a range of 36.5°C–37.5°C using a homothermic heating pad during surgery. Briefly, a polysiloxane‐coated monofilament was inserted into the internal carotid artery to occlude the middle cerebral artery. After 30 min, the monofilament was withdrawn to initiate the reperfusion process. After surgery, the incision site was disinfected with iodophor, and mice were placed in a 30°C incubator until fully conscious to maintain a stable body temperature. A total nutritional jelly was provided to support their energy supply. The sham‐operated group underwent the same surgical procedure as described above but without MCAO/R. In a separate group, Rg1 was administered intraperitoneally at 30 mg/kg/day, starting three days before surgery and continuing on the day of the procedure. After 24 h of reperfusion, cerebral blood flow in the three groups was assessed using a Laser Scattering Blood Flow Instrument (RWD Life Sciences, Shenzhen, China).

### 2.13 Neurologic Deficits Evaluation

After a 24‐h interval following MCAO/R, the neurological status of the mice was detected using a standardised scoring system to evaluate the extent of neurologic deficits [8]. The scoring system ranges from 0 (normal) to 12 (maximum deficits). A score greater than 1 indicates successful induction of a focal cerebral ischaemic event.

### 2.14 Behavioural Tests

The rotarod test was employed to assess motor coordination and balance in mice following MCAO/R surgery. Mice were positioned on a rod that accelerated from 4 to 30 rpm over 4 min. The latency for each mouse to lose balance and fall was recorded. Before MCAO/R surgery, mice were assigned to different groups and underwent three daily training sessions for three consecutive days to establish baseline performance. After 24 h post‐MCAO/R surgery, each mouse underwent three trials on the rotarod to assess motor capabilities. The adhesive‐removal test was performed to assess sensory function by measuring the time it takes for mice to remove a small adhesive dot from their forepaws. After acclimating to the testing environment, two adhesive dots were placed on the mice's paws. The time taken for the mice to remove the dots with their teeth was recorded, with a maximum allowed time of 2 min per dot. Prior to surgery, mice underwent three acclimation sessions per day for 3 days to become familiar with the test conditions. At 24 h post‐MCAO/R, the test was conducted to evaluate the mice's sensory and motor responsiveness.

### 2.15 Cerebral Infarction Rate Determination

Cerebral infarction following MCAO/R surgery was assessed using 2,3,5‐triphenyltetrazolium chloride (TTC) staining (Sigma‐Aldrich, Missouri, USA). Brains were rapidly removed, frozen, and sliced into six 2‐mm‐thick sections. These sections were immersed in 3% TTC solution at 37°C for 20 min and then fixed in 4% PFA solution. Infarcted regions appeared white, in contrast to the red healthy tissue. The extent of ischemic injury was quantified by capturing images of the coronal slices and analysing them with ImageJ software. To address potential overestimation of infarct volume due to cerebral oedema, we implemented a standardised correction formula in our analysis: Corrected Infarct Volume = Infarct Volume (∑infarct area × slice thickness) × (Contralateral Hemisphere Area/Ipsilateral Hemisphere Area).

### 2.16 Pathological Tissue Staining

H&E staining was employed to observe the general tissue structure, TUNEL staining to detect apoptotic cells, and Nissl staining to identify the distribution of Nissl bodies within neurons. Briefly, brain samples were fixed in 4% PFA solution and then embedded in paraffin. Sections, 5 μm thick, were prepared and stained with H&E (G1076‐500ML), TUNEL (G1501‐50 T) and Nissl (G1036‐100ML).

### 2.17 Immunohistochemistry (IHC)

Brain tissue samples were fixed, embedded and sectioned coronally at 5‐μm slices. The sections were incubated overnight at 4°C with primary antibodies against P62 (1:400), MFN1 (1:100) and MFN2 (1:200). Subsequently, sections were incubated with HRP‐conjugated secondary antibodies. Colour development was performed using a 3,3′‐diaminobenzidine (DAB) kit (Servicebio, China), followed by haematoxylin counterstaining. The entire ipsilateral infarct area was analysed and quantified using ImageJ software.

### 2.18 Data Analysis

Statistical analysis was performed using GraphPad Prism (6.0). All data were collected from at least three independent experiments and are presented as mean ± standard deviation (SD). The Shapiro–Wilk test was conducted to evaluate the normality of the data. For data sets exhibiting normal distribution, statistical analyses were conducted as follows: One‐way ANOVA with Dunnett's post hoc test. Two‐way ANOVA with Tukey's post hoc test. Statistical significance was set at a *p*‐value of less than 0.05.

## 3 Results

### 3.1 Ginsenoside Rg1 Alleviates CIRI In Vitro

**FIGURE 1:**
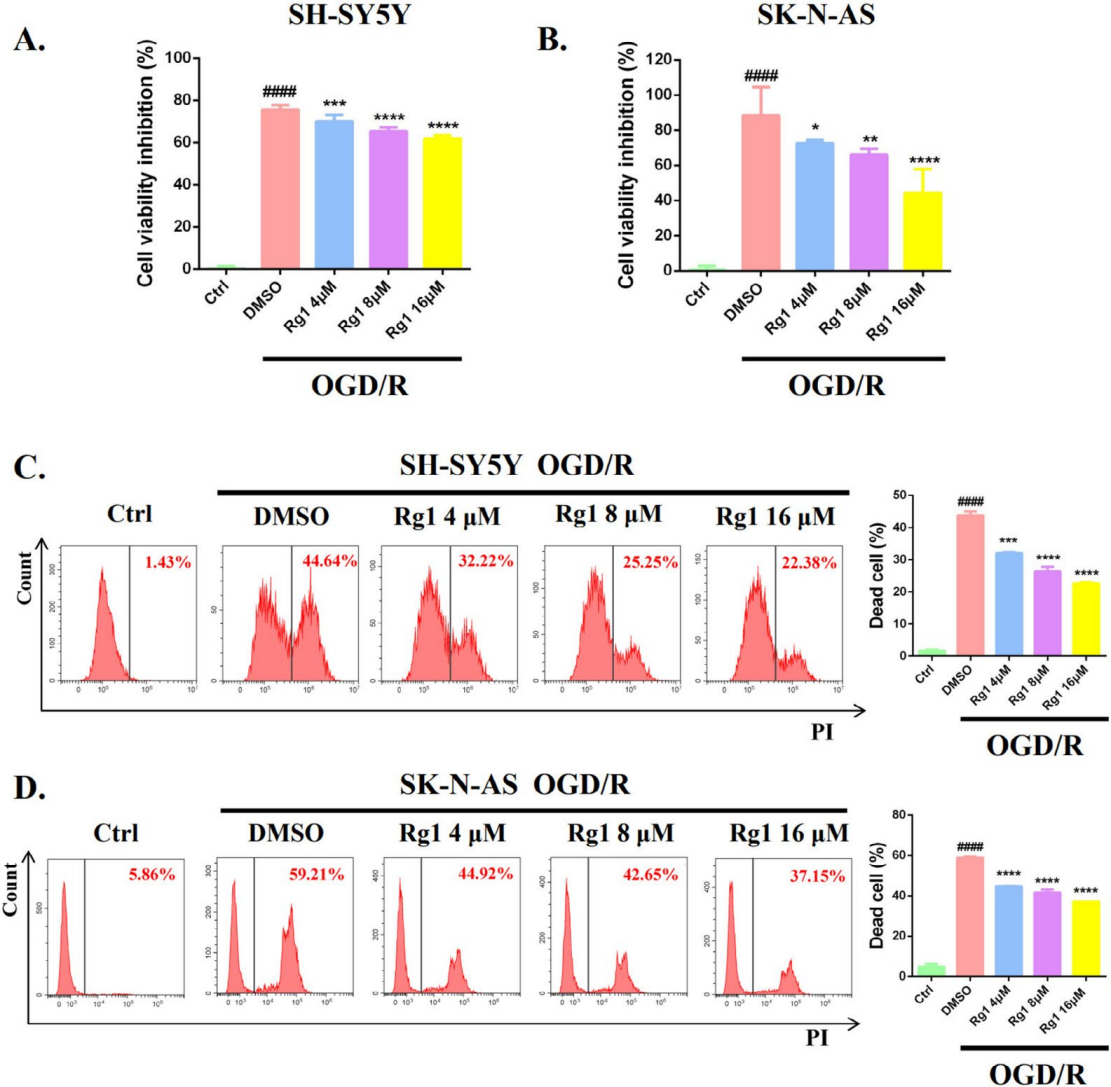
Rg1 protects against CIRI in vitro. The SH‐SY5Y cells were exposed to OGD for 10 h and SK‐N‐AS cells for 12 h, followed by a 24‐h reperfusion period with varying concentrations of Rg1 (4, 8 and 16 μM). (A, B) The effect of Rg1 on cell viability was assessed via CCK‐8 assay. (C,D) Representative flow cytometry images showing cell death detected by the PI exclusion assay, with quantification of cell death ratio displayed on the right. *p < 0.05; **p < 0.01; ***p < 0.001; ****p < 0.0001 compared to DMSO group. In addition, ^####^ *p* < 0.0001 compared to DMSO group and Ctrl group (cells kept under normal culture conditions).

OGD/R, a condition that mimics CIRI, was induced in SH‐SY5Y and SK‐N‐AS cells to evaluate the protective effect of Rg1. The results, as illustrated in Figure 1A,B, demonstrated that exposure to OGD/R conditions significantly reduced cell viability by approximately 80% compared to the control group. However, treatment with Rg1 at varying concentrations (4, 8, and 16 μM) led to a dose‐dependent reduction of the cytotoxic effects induced by OGD/R. This protective effect was further validated by the PI exclusion assay, which assesses cell membrane integrity and indicates cell death. OGD/R caused a 45%–60% increase in cell death compared to the control group, whereas Rg1 treatment significantly reduced the death ratio by more than 20% (Figure 1C,D). These findings underscore the potent protective role of Rg1 against CIRI in vitro.

### 3.2 Rg1 Inhibits Mitophagy and Protects Mitochondrial Function During OGD/R

**FIGURE 2:**
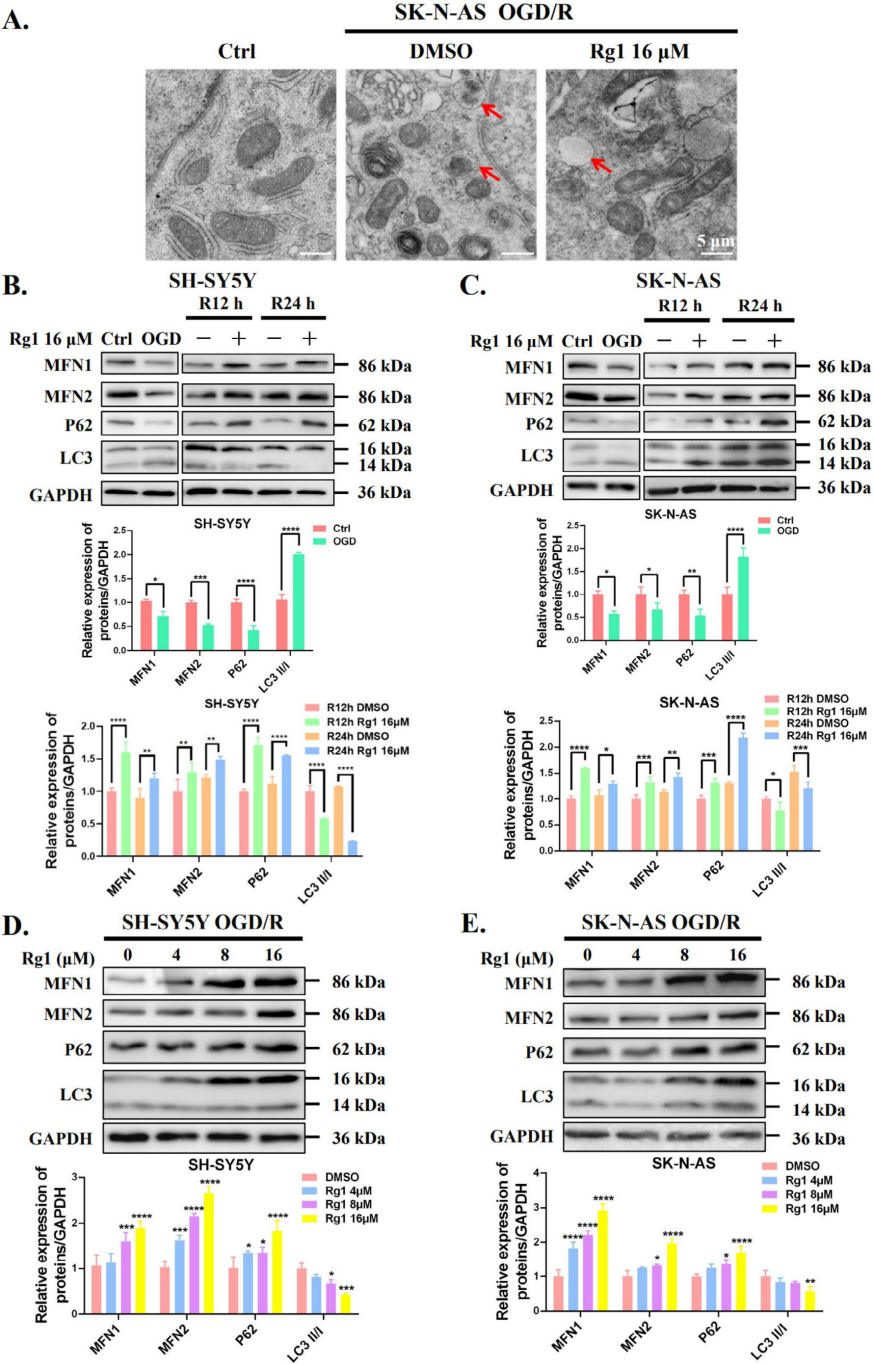
Inhibitory effect of Rg1 on mitophagy in OGD/R cells. (A) Representative transmission electron microscopy images showing mitochondrial ultrastructure in SK‐N‐AS cells after 12 h OGD and 24 h reperfusion, with or without 16 μM Rg1 treatment. Red arrows indicate double membrane vesicles with or without enclosed mitochondria (Scale bar: 5 μm). (B, C) Western blotting analysis of MFN1, MFN2, P62 and LC3 protein levels in SH‐SY5Y and SK‐N‐AS cells subjected to OGD followed by 12 or 24 h of reperfusion, with or without 16 μM Rg1 treatment. GAPDH served as the internal loading control. (D, E) The indicated proteins were evaluated in OGD‐exposed SH‐SY5Y and SK‐N‐AS cells after 24 h of reperfusion, with or without Rg1 treatment. Band quantification was performed using ImageJ software. *p < 0.05; **p < 0.01; ***p < 0.001; ****p < 0.0001 versus DMSO group or indicated group.

Recognising the importance of mitophagy in CIRI, we investigated its role in Rg1's protective effect [21]. During cellular stress, autophagosomes engulf damaged mitochondria, forming mitophagosomes that merge with lysosomes for degradation, a key step in mitophagy [22]. Using transmission electron microscopy, we observed that mitochondria in the control group displayed a healthy tubular morphology with well‐defined cristae. In contrast, mitochondria in the OGD/R group appeared elliptical with reduced cristae, indicating mitochondrial damage. Furthermore, OGD/R increased the presence of elliptical mitochondria within double membrane vesicles, while Rg1 treatment significantly reduced this occurrence (Figure 2A). These findings suggest that Rg1 may modulate mitophagy during the OGD/R phase.

Excessive mitophagy typically reduces mitochondrial quantity, leading to a depletion of mitochondrial proteins, including mitochondrial fusion proteins 1 (MFN1) and 2 (MFN2) [23]. As depicted in Figure 2B,C, MFN1 and MFN2 levels significantly decreased during OGD, with partial restoration upon reperfusion, indicating dynamic changes in mitophagy. Rg1 treatment increased MFN1 and MFN2 levels compared to the untreated group after 12 and 24 h of reperfusion, suggesting that Rg1 inhibits mitophagy. Furthermore, OGD elevated the LC3‐II/LC3‐I ratio and reduced P62 protein levels, both markers of autophagy. During reperfusion, Rg1 increased the levels of P62 and decreased the LC3‐II/LC3‐I ratio, indicating inhibition of mitophagy flux. Consistently, Rg1 increased the protein levels of MFN1, MFN2 and P62, decreased the LC3‐II/LC3‐I ratio in a dose‐dependent manner (Figure 2D,E). These results suggest that Rg1 exerts its protective effect by suppressing OGD‐induced mitophagy in SH‐SY5Y and SK‐N‐AS cells.

Since mitophagy is indispensable for mitochondrial quality control, our study explored the effect of Rg1 on mitochondrial function. OGD/R caused mitochondrial dysfunction, as evidenced by reduced mitochondrial membrane potential and increased ROS levels. In contrast, Rg1 treatment effectively maintained the mitochondrial membrane potential and reduced mtROS accumulation, without affecting ATP production (Figures S1–2). These findings suggest that Rg1 exerts a protective effect on mitochondrial function, potentially through the inhibition of mitophagy as a regulatory mechanism.

### 3.3 The Inhibition of Mitophagy Is Crucial for Rg1's Neuroprotection Against CIRI

**FIGURE 3:**
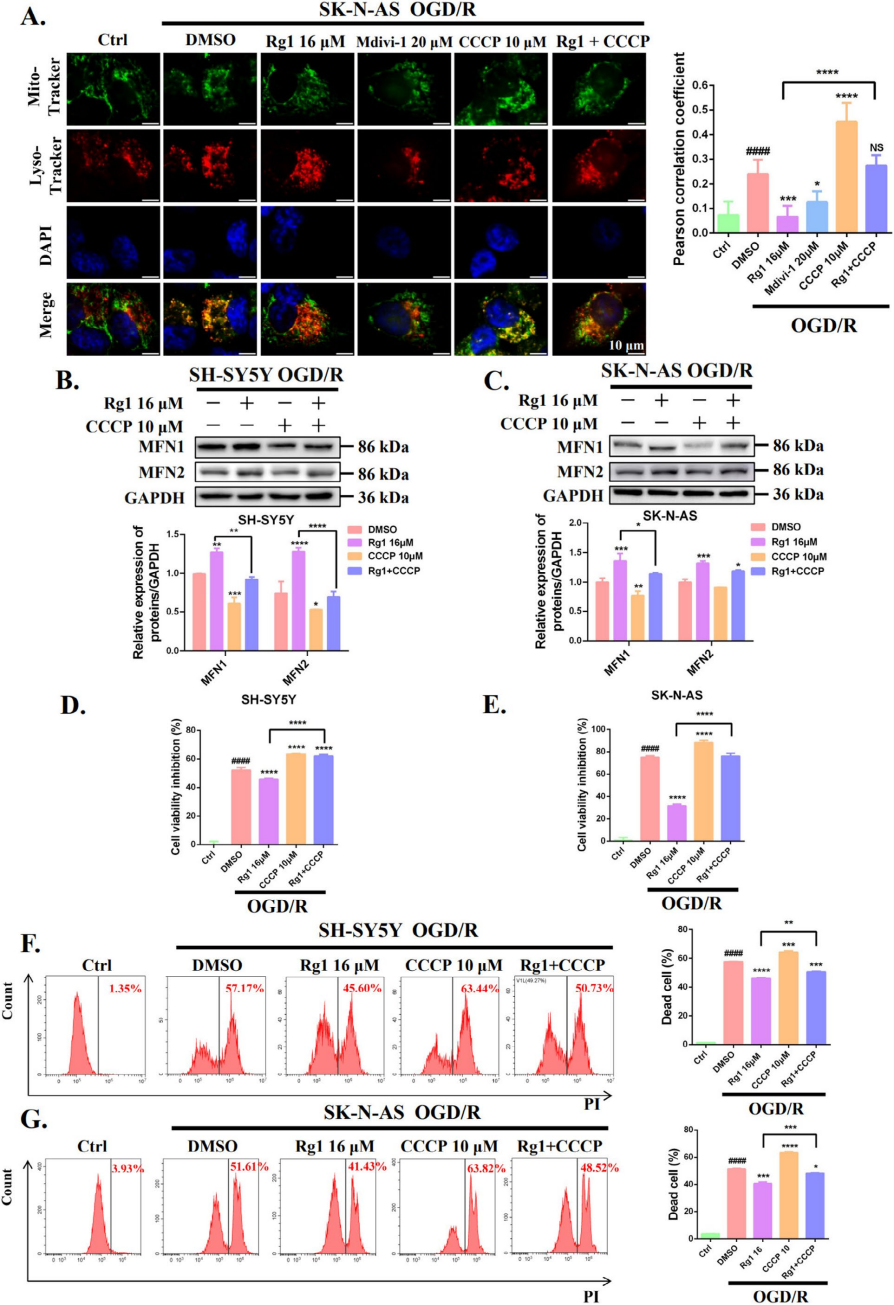
Neuroprotective effect of ginsenoside Rg1 are mediated through the regulation of mitophagy. (A) SK‐N‐AS cells subjected to OGD/R were treated with DMSO, 16 μM Rg1, 20 μM Mdivi‐1 (a mitophagy inhibitor), 10 μM CCCP (a mitophagy inducer), or a combination of CCCP and Rg1. The immunofluorescence assay showed co‐localisation of mitochondria (MitoTracker, green) and lysosomes (LysoTracker, red), with DAPI (blue) highlighting the cell nuclei (Scale bar: 10 μm). (B, C) SH‐SY5Y and SK‐N‐AS cells subjected to OGD followed by 24 h of reperfusion were treated with the indicated substances. The expression levels of MFN1 and MFN2 were analysed by Western blotting. Cell viability inhibition under various treatment conditions was evaluated using the CCK‐8 assay (D, E), and the ratio of cell death was determined by the PI exclusion assay (F, G). ^####^ *p* < 0.0001 versus Ctrl group. *p < 0.05; **p < 0.01; ***p < 0.001; ****p < 0.0001 versus DMSO group or indicated group. “NS” indicates no significant difference.

A critical event in mitophagy is the fusion of mitophagosomes with lysosomes, which enables the clearance of dysfunctional mitochondria [22]. To assess whether Rg1 treatment hinders this fusion process, we analysed the spatial relationship between mitochondria (MitoTracker) and lysosomes (LysoTracker). Immunofluorescent analysis showed that OGD/R treatment altered mitochondrial morphology from filamentous to punctate and increased the co‐localisation of mitochondria and lysosomes compared to untreated cells. This effect was similar to that of the mitophagy inducer CCCP. In contrast, treatment with Rg1 or Mdivi‐1 (a mitophagy inhibitor) significantly improved mitochondrial morphology in OGD/R cells and notably reduced the co‐localisation of mitochondria and lysosomes. Moreover, co‐treatment with CCCP and Rg1 impaired mitochondrial morphology and increased the co‐localisation of mitochondria and lysosomes compared to Rg1 treatment alone (Figure 3A). As expected, the MFN1 and MFN2 levels were upregulated by Rg1, whereas CCCP reduced these levels in OGD/R cells (Figure 3B,C). These results underscore that Rg1 may protect mitochondrial function by inhibiting mitophagy, specifically by disrupting the fusion of mitochondria and lysosomes under OGD/R conditions.

Additionally, we evaluated whether prolonged Rg1 treatment (48 h or 96 h) impairs mitochondrial function and induces cytotoxicity in OGD/R SH‐SY5Y and SK‐N‐AS cells. The results demonstrated that Rg1 alleviated OGD/R‐induced cytotoxicity and preserved mitochondrial membrane potential in a dose‐dependent manner, indicating that long‐term mitophagy inhibition by Rg1 did not elicit significant detrimental effects (Figure S3–4). Furthermore, we investigated whether Rg1's inhibition of mitophagy is crucial for its neuroprotective effects. Compared to the OGD/R model group, administration of CCCP at the onset of reperfusion further reduced cell viability and exacerbated cell death. These findings suggest that excessive mitophagy activation during reperfusion contributes to CIRI (Figure 3D–G). Moreover, co‐administration of CCCP with Rg1 significantly diminished Rg1's ability to improve cell viability and reduce cell death in OGD/R‐treated SH‐SY5Y and SK‐N‐AS cells. Taken together, these findings suggest that Rg1's neuroprotective effects are at least partly due to its ability to inhibit mitophagy.

### 3.4 Rg1 Disrupts Mitophagosomes‐Lysosomes Fusion, Leading to the Accumulation of Mitophagy‐Initiating Proteins

**FIGURE 4:**
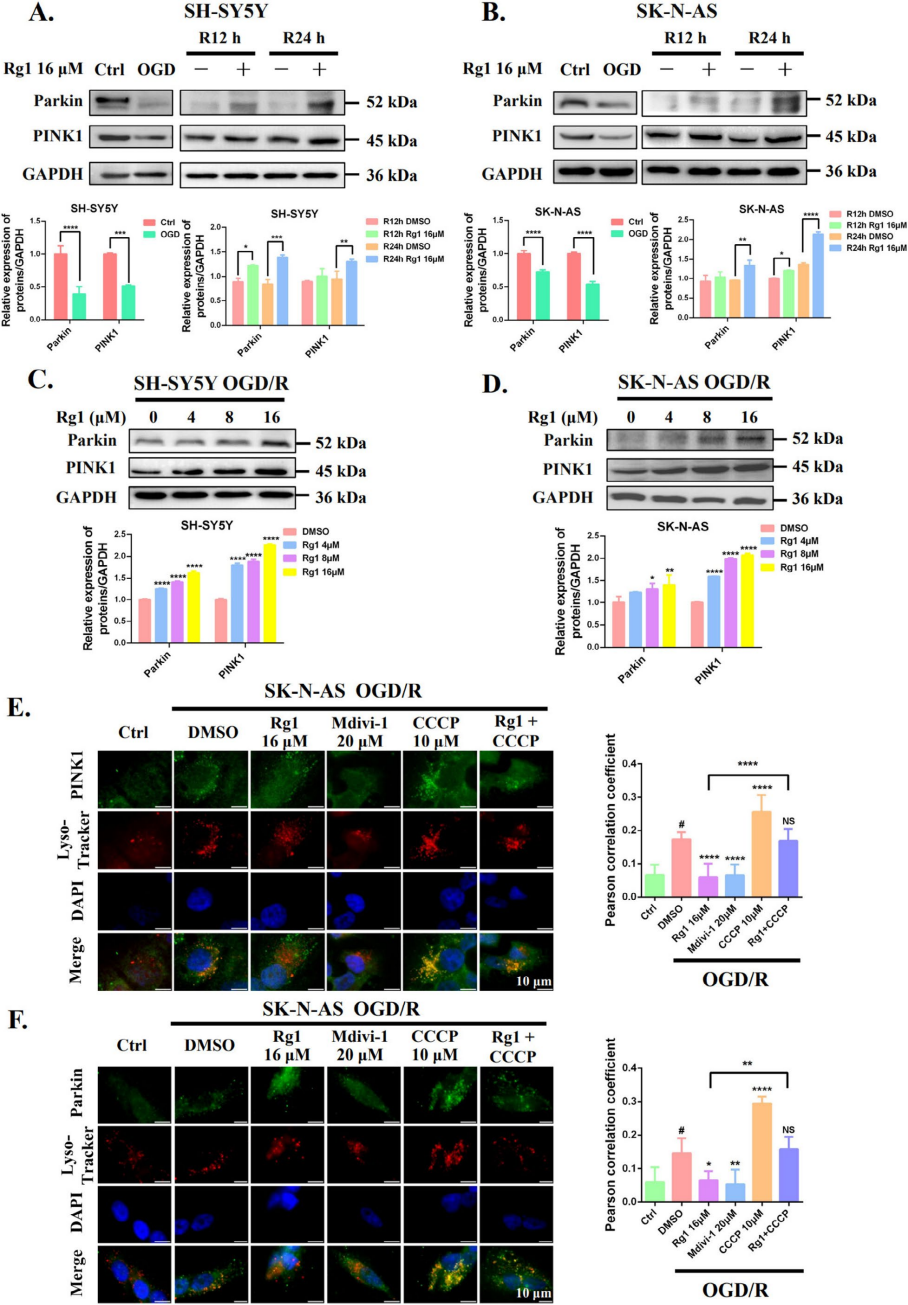
Rg1 disrupts mitophagosomes‐lysosomes fusion, leading to PINK1 and Parkin protein accumulation. (A, B) SH‐SY5Y and SK‐N‐AS cells underwent OGD followed by 12 h or 24 h of reperfusion with or without Rg1 (16 μM) treatment. Western blotting was utilised to assess PINK1 and Parkin levels which were quantified using ImageJ software. (C, D) The effects of varying Rg1 concentrations (0 to 16 μM) on PINK1 and Parkin expressions in OGD/R cells were examined. (E, F) SK‐N‐AS cells were treated with DMSO, Rg1 (16 μM), Mdivi‐1 (20 μM), CCCP (10 μM) or a combination of Rg1 and CCCP. Representative images of immunofluorescence show PINK/Parkin (green) and lysosomes (LysoTracker, red), with DAPI (blue) marking the cell nuclei (Scale bar: 10 μm). Statistical significance is indicated as follows: *p < 0.05; **p < 0.01; ***p < 0.001; ****p < 0.0001 when compared to the DMSO group. ^#^ *p* < 0.05 when compared to the Ctrl group. “NS” indicates no significant difference.

The PINK1/Parkin signalling pathway plays a crucial role in mitochondrial physiology, particularly in initiating ubiquitin‐dependent mitophagy [14, 24, 25]. We investigated whether Rg1 inhibits the initiation of mitophagy by modulating PINK1 and Parkin in OGD/R‐treated SH‐SY5Y and SK‐N‐AS cells. Our results showed a transient reduction in both proteins during OGD, followed by partial recovery during reperfusion. Notably, Rg1 treatment significantly upregulated PINK1 and Parkin levels in a time‐ and dose‐dependent manner in OGD/R‐exposed SH‐SY5Y and SK‐N‐AS cells (Figure 4A–D). Give that Rg1 impedes the fusion of mitochondria and lysosomes, we further explored whether the increased PINK1 and Parkin levels were linked to an accumulation of damaged mitochondria that were not efficiently degraded by lysosomes. Immunofluorescence staining revealed enhanced co‐localisation of PINK1 and Parkin with lysosomes following OGD/R treatment. This co‐localisation was significantly reduced by Rg1 and the mitophagy inhibitor Mdivi‐1. In contrast, CCCP significantly promoted this co‐localisation and negated Rg1's inhibitory effect when co‐administered (Figure 4E,F). Additionally, CCCP markedly decreased PINK1 and Parkin protein levels, counteracting the Rg1‐induced increase in these proteins (Figure 6A,B).

**FIGURE 5:**
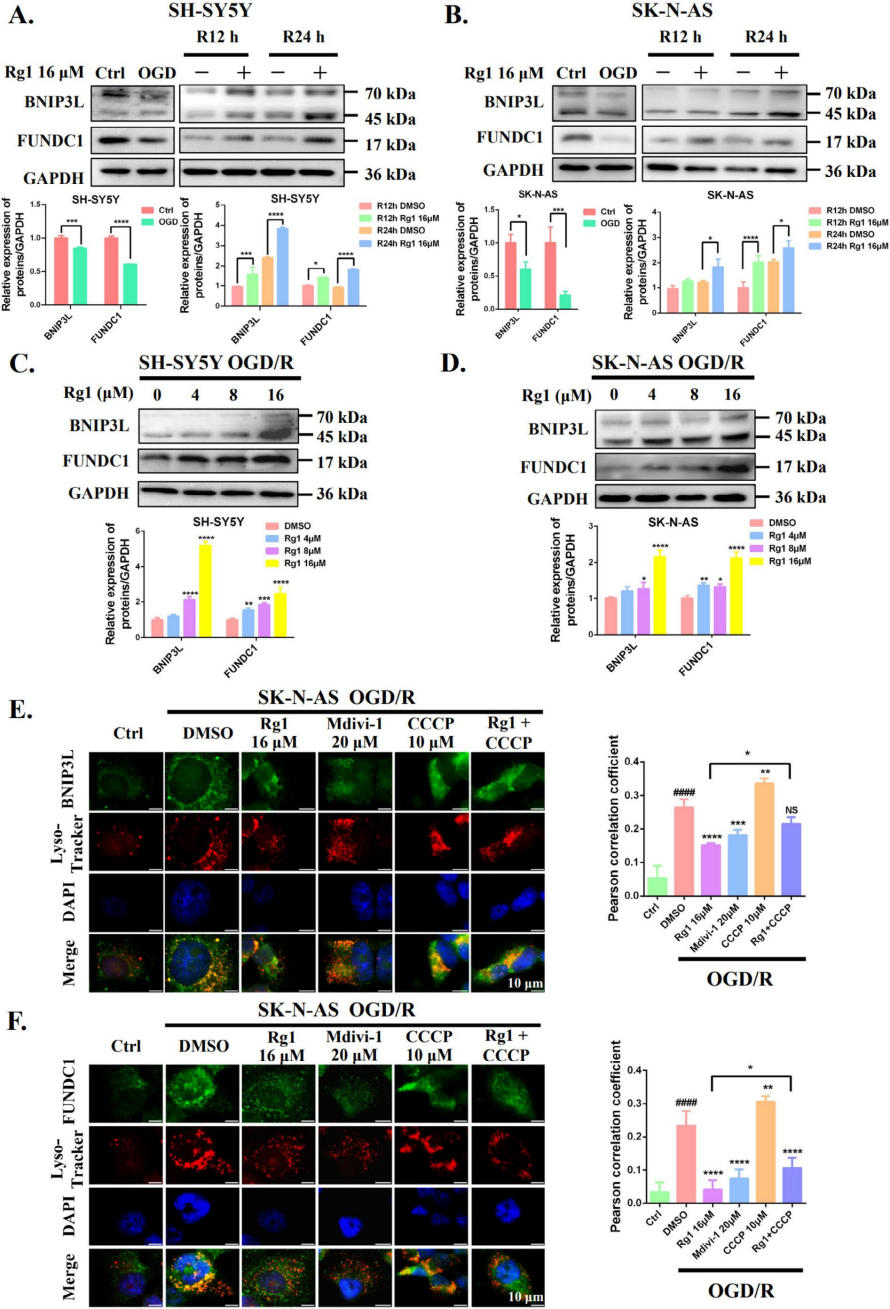
Rg1 disrupts mitophagosomes‐lysosomes formation, resulting in BNIP3L and FUNDC1 accumulation. (A, B) SH‐SY5Y and SK‐N‐AS cells underwent OGD followed by 12 h or 24 h of reperfusion with or without Rg1 (16 μM) treatment. Western blotting was used to assess BNIP3L and FUNDC1 levels which were quantified using ImageJ software. (C, D) The effects of varying Rg1 concentrations (0–16 μM) on BNIP3L and FUNDC1 expression in OGD/R cells were examined. (E, F) SK‐N‐AS cells were treated with DMSO, Mdivi‐1 (20 μM), Rg1 (16 μM), CCCP (10 μM) or a combination of Rg1 and CCCP. Representative images of immunofluorescence show BNIP3L or FUNDC1 (green) and lysosomes (LysoTracker, red), with DAPI (blue) marking the cell nuclei (Scale bar: 10 μm). *p < 0.05; **p < 0.01; ***p < 0.001; ****p < 0.0001 compared with DMSO group. ^####^ *p* < 0.0001 compared to Ctrl group. “NS” indicates no significant difference.

**FIGURE 6:**
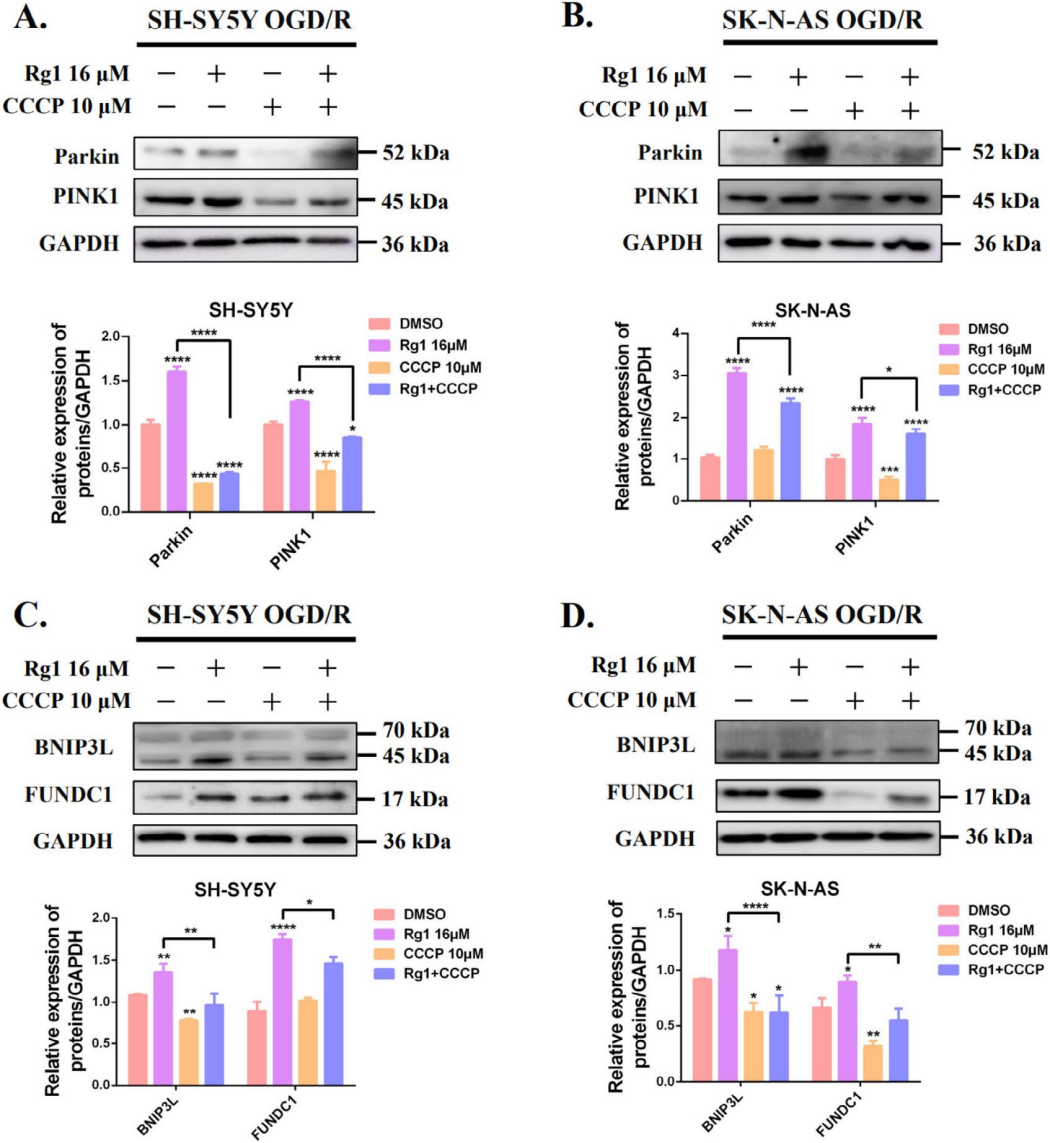
Rg1 (A, B) Western blotting analyses of PINK1 and Parkin protein levels under indicated treatments in OGD/R SH‐SY5Y and SK‐N‐AS cells. (C, D) Western blotting analyses of BNIP3L and FUNDC1 protein levels under indicated treatments in OGD/R SH‐SY5Y and SK‐N‐AS cells. GAPDH served as the internal loading control. Statistical significance is indicated as follows: *p < 0.05; **p < 0.01; ***p < 0.001; ****p < 0.0001 when compared to the DMSO group. ^#^ *p* < 0.05 when compared to the Ctrl group. “NS” indicates no significant difference.

Furthermore, we also investigated whether Rg1's inhibition of mitophagy involves ubiquitin‐independent pathways regulated by the outer mitochondrial membrane proteins BNIP3L and FUNDC1 [26]. Rg1 treatment upregulated BNIP3L and FUNDC1 in a time‐ and dose‐dependent manner (Figures 5A–D and 6C,D), but this effect was abolished when combined with CCCP. Additionally, Rg1 and Mdivi‐1 significantly reduced the co‐localisation of BNIP3L and FUNDC1 with lysosomes, which was increased by OGD/R in SK‐N‐AS cells. CCCP reversed Rg1's inhibitory effects on co‐localisation and also decreased the elevated levels of BNIP3L and FUNDC1 induced by Rg1 (Figure 5E,F). These findings suggest that Rg1's inhibitory effect on mitophagy is primarily due to its disruption of mitophagosome‐lysosome fusion, rather than modulation of mitophagy initiation pathways mediated by PINK1/Parkin, BNIP3L or FUNDC1.

### 3.5 Rg1 Mitigates CIRI and Inhibits Mitophagy in MCAO/R Mice

**FIGURE 7:**
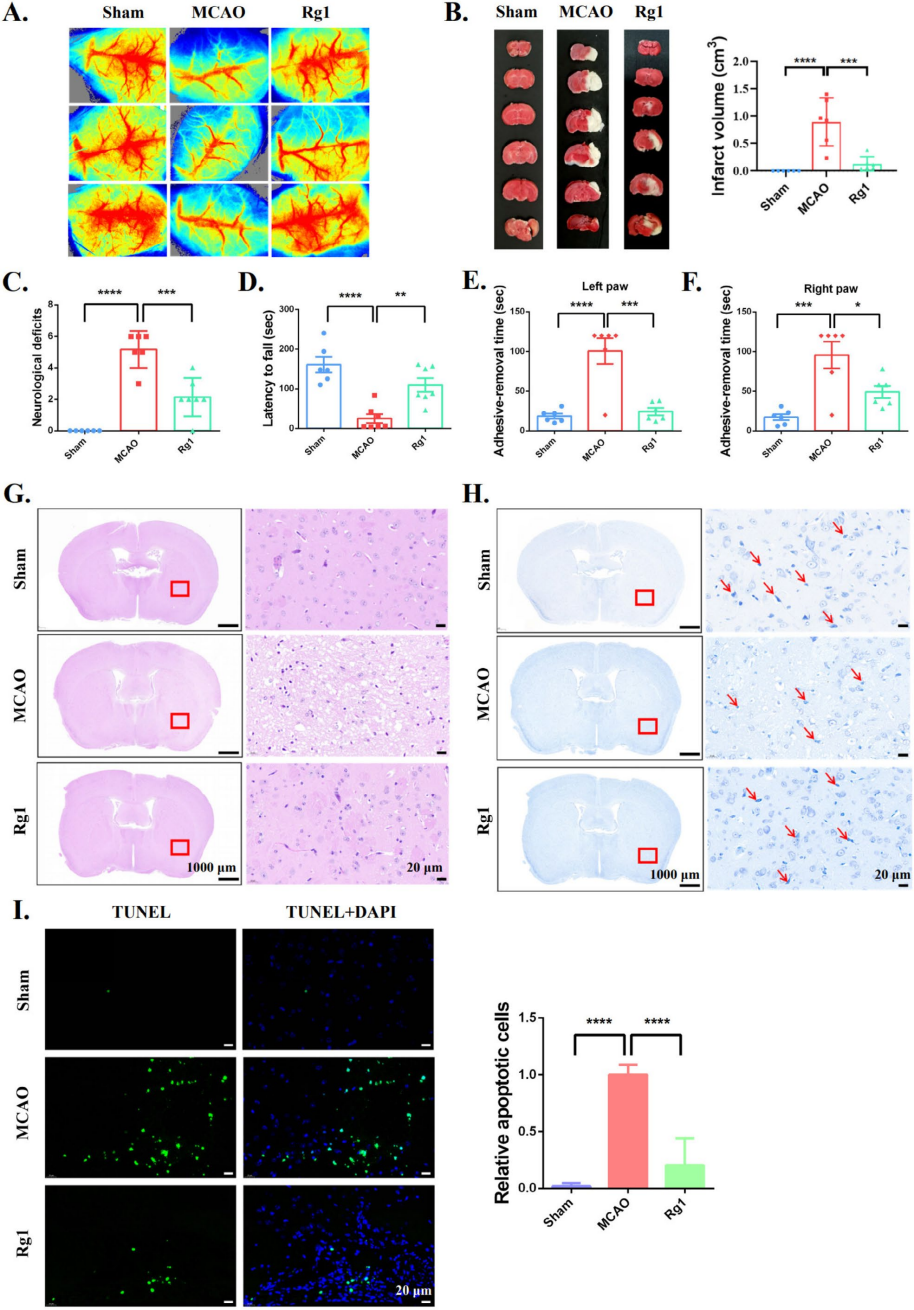
Rg1's neuroprotective effect against CIRI in vivo. Mice received Rg1 intraperitoneally at a daily dose of 30 mg/kg, starting three days before the MCAO/R surgery and continuing on the day of the procedure. The surgery involved 30 min of ischemia followed by 24 h of reperfusion. (A) Representative images show brain blood flow in the different treatment groups. (B) Representative images of TTC staining, with quantitative analysis of the infarcted areas performed using ImageJ software. (C) Neurological deficit scores, (D) Rotarod test, and (E, F) Adhesive‐removal test was performed to evaluate the protective effect of Rg1 against CIRI in mice. (G, H) Histological analysis of brain tissue was preformed using H&E staining (Scale bar: 1000 μm and 20 μm) and Nissl staining to evaluate neuronal cells (Scale bar: 1000 μm and 20 μm). Red arrows highlight the Nissl bodies. (I) TUNEL assay of brain sections was used to identify apoptotic cells (green), with quantification of the relative number of apoptotic cells shown on the right (Scale bar: 20 μm). *p < 0.05; **p < 0.01; ***p < 0.001; ****p < 0.0001 compared to the indicated group.

We employed an MCAO/R mouse model to assess the neuroprotective effects of Rg1 against CIRI in vivo. Following MCAO/R surgery, significant occlusion of cerebral blood flow was observed in MCAO/R mice. However, Rg1 treatment markedly restored blood reperfusion (Figure 7A). Additionally, Rg1 significantly reduced the white infarct volume in the brain tissue caused by MCAO/R (Figure 7B). Rg1 also improved neurological impairments and enhanced behavioural outcomes, as evidenced by better motor coordination on the rotarod and improved sensory function in the adhesive‐removal tests (Figure 7C–F). Histopathological analysis revealed disorganised brain tissue in the MCAO/R group, characterised by vacuolisation, shrinkage and structurally deficient neuronal cells with pale or lysed Nissl body staining. Rg1 treatment attenuated these pathological changes and significantly enhanced neuronal survival (Figure 7G,H). Additionally, Rg1 reduced MCAO/R‐induced apoptotic cells in brain tissue by approximately 70% compared to the untreated MCAO/R group (Figure 7I). In conclusion, ginsenoside Rg1 exhibits cerebroprotective effects against CIRI and may serve as a promising adjunct for vascular recanalisation.

**FIGURE 8:**
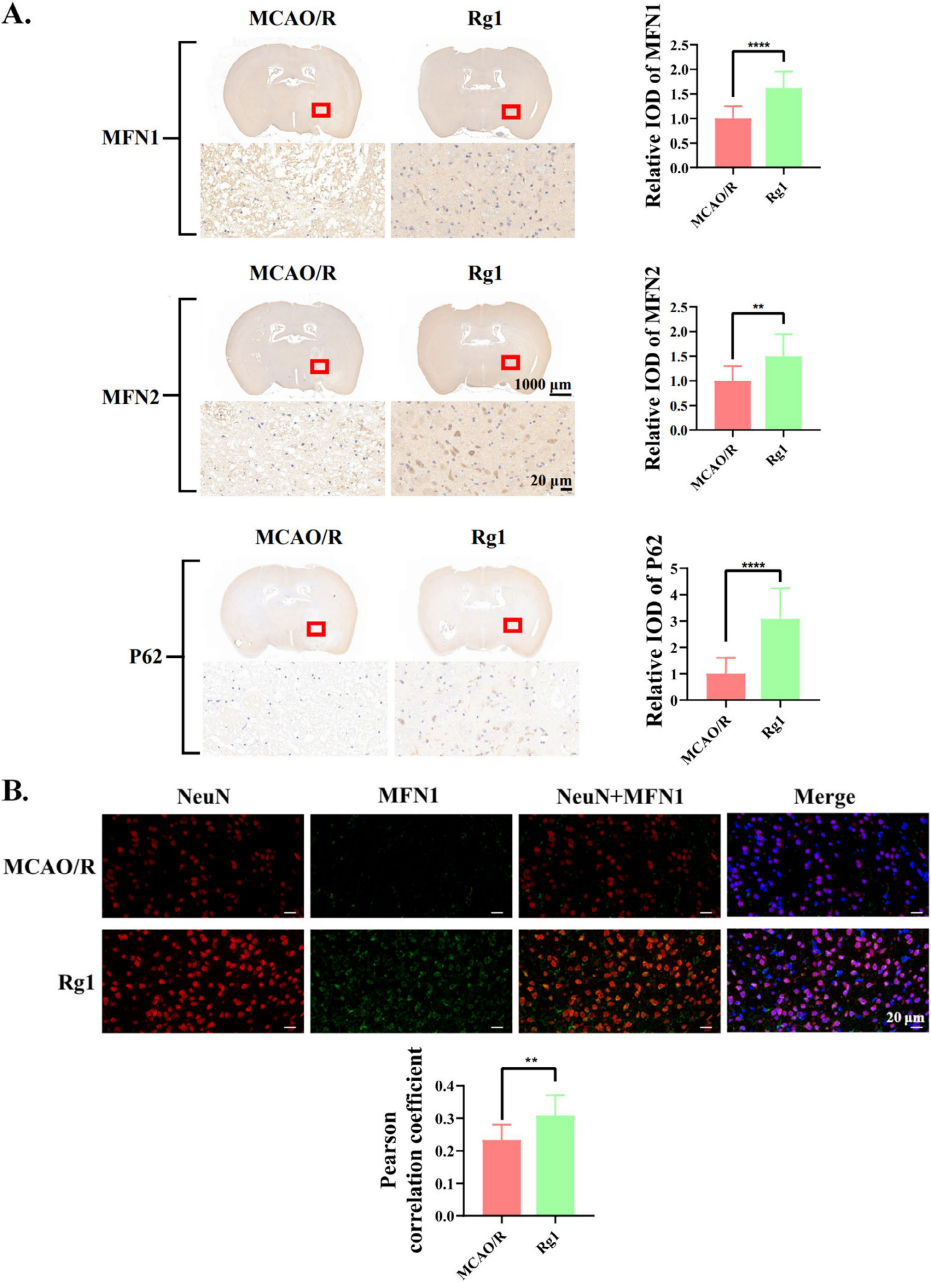
Inhibitory effect of Rg1 on mitophagy in MCAO/R mice. (A) IHC analysis of MFN1, MFN2 and P62 expression in brain tissues from MCAO/R and Rg1‐treated groups. Representative images and quantification of integrated optical density show protein expression in the entire ipsilateral infarct area. Scale bars: 1000 μm and 20 μm. (B) Representative images of double immunofluorescence staining of NeuN (neuronal marker, red) and MFN1 (mitophagy marker, green), with co‐localisation quantitative analysis. DAPI (blue) was used to indicate cell nuclei. Scale bar: 20 μm. All results are presented as mean ± SD and were replicated at least three times. Statistical significance was indicated as follows: **p < 0.01; ****p < 0.0001 compared to the MCAO/R group.

To investigate the role of Rg1 in modulating mitophagy in vivo, we performed IHC on mouse brain tissue to analyse mitophagy‐related markers (P62, MFN1 and MFN2) after MCAO/R injury. Quantitative analysis of the ipsilateral infarct region revealed that Rg1 significantly upregulated the expression of P62, MFN1 and MFN2 compared to the MCAO/R group (Figure 8A). Furthermore, immunofluorescence analysis showed that Rg1 treatment significantly increased MFN1 expression within NeuN‐positive neurons (Figure 8B), suggesting that its neuroprotective effects may arise from the suppression of neuronal mitophagy. These findings suggest that Rg1 inhibits mitophagy in vivo, consistent with in vitro findings.

## 4 Discussion

Ginsenoside Rg1 has garnered significant attention for its potential neuroprotective effects, particularly in mitigating CIRI [6]. Our research, employing both in vitro (OGD/R) and in vivo (MCAO/R) models, further substantiates the therapeutic efficacy of Rg1 in reducing the harmful effects of CIRI. These findings suggest that Rg1 could be a viable candidate for the development of stroke therapeutics. Future research, including a deeper exploration of its molecular mechanisms and clinical trials, is essential to fully realise Rg1's therapeutic potential in stroke management.

Extensive research has explored the role of mitophagy, with evidence indicating that its dysregulation can exacerbate mitochondrial dysfunction and neuronal damage following CIRI injury [27]. Our findings revealed that enhanced mitophagy, induced by CCCP, worsened CIRI in our OGD/R model. This exacerbation was evidenced by increased inhibition of cellular viability and higher rates of cell death, suggesting that an excessive mitophagic response contributes to CIRI. Previous studies have demonstrated Rg1's ability to modulate mitophagy, such as by activating the PINK1/Parkin pathway to ameliorate Alzheimer's disease [28] or by providing protection against nutritional stress in H9c2 cells via the AMPK/PINK1 signalling pathway [29]. In this study, we uncovered that Rg1 mitigates CIRI by suppressing mitophagy. Mechanistically, Rg1 reduced the number of autophagosomes enclosing mitochondria, enhanced the levels of mitochondria‐associated proteins including MFN1, MFN2 and P62, and impeded the fusion process between mitochondria and lysosomes. Importantly, the neuroprotective effect of Rg1 was diminished by CCCP, highlighting the importance of mitophagy inhibition in Rg1's neuroprotective mechanism.

A crucial step in mitophagy is the fusion of mitophagosomes with lysosomes, a process essential for the degradation and recycling of damaged mitochondria. This fusion is vital for maintaining neuronal function and survival during CIRI [30]. Our study provides evidence that Rg1 treatment markedly inhibited the co‐localisation of mitochondria and lysosomes, leading to an accumulation of mitochondria. These findings suggest that Rg1 impedes the late stage of mitophagy, thereby preventing excessive mitophagy during the I/R phase. Interestingly, our previous study demonstrated that ginsenoside Rg1 inhibits autophagy by disrupting lysosomal function, without affecting the fusion of autophagosomes with lysosomes [8]. These findings suggest that Rg1 may modulate autophagy and mitophagy through diverse mechanisms, highlighting the need for further investigation.

We further investigated whether Rg1 inhibits the initiation of mitophagy by suppressing the activation of key pathways, such as the PINK1/Parkin pathway, BNIP3L or FUNDC1, which are known to trigger mitophagy during reperfusion [31, 32, 33]. Notably, our findings revealed that these mitophagy‐initiating proteins were not downregulated by Rg1 but rather increased. This increase was reversed by treatment with CCCP, which also decreased the expression of these proteins. Given that CCCP enhanced the co‐localisation of these proteins with lysosomes, while Rg1 and Mdivi‐1 reduced this co‐localisation, we speculated that the increase in mitophagy‐initiating proteins observed with Rg1 is due to the accumulation of mitochondria. Since proteins such as Rab7 and TBC1D15 were reported to facilitate the fusion of mature mitophagosomes with lysosomes [34]. Further investigation into the impact of Rg1 on Rab7 and TBC1D15 is warranted to elucidate its role in inhibiting the fusion between mitophagosomes and lysosomes.

In the present study, our in vitro data demonstrated that Rg1 effectively inhibited excessive mitophagy when administered at reperfusion onset. However, translating this protocol to in vivo setting is challenging due to delayed drug distribution: intraperitoneal injection upon reperfusion initiation fails to achieve sufficient brain drug concentrations within the critical therapeutic window. To address this limitation, we employed a pre‐treatment strategy to ensure adequate tissue drug levels during reperfusion, as previously reported [35, 36, 37, 38, 39]. Mice were pre‐treated with Rg1 for 3 days before reperfusion, achieving therapeutic drug levels in the brain during the ischemic phase. Despite variations in treatment timelines, these findings consistently support the neuroprotective effects of Rg1 in vivo. Nevertheless, the optimal timing for Rg1 administration to maximise therapeutic efficacy remains undetermined. These gaps highlight the urgent need for pharmacokinetic/pharmacodynamic (PK/PD) studies to determine Rg1's half‐life in the reperfusion‐injured brain and to establish optimal dosing windows [40]. Such investigations are critical for translating Rg1 into clinically viable interventions that balance practicality and efficacy.

In conclusion, our study elucidates the neuroprotective mechanisms of Rg1 against CIRI, emphasising its ability to inhibit mitophagy. Specifically, Rg1 impedes the fusion of mitophagosomes with lysosomes, thereby preventing mitochondrial degradation, rather than directly inhibiting the initiation of mitophagy. These insights enhance our understanding of the pathophysiological processes underlying CIRI and suggest that Rg1 could be a promising therapeutic candidate. The potential of Rg1 to mitigate CIRI by regulating mitophagy opens new avenues for further research and clinical exploration.

## Author Contributions

**A.L.:** investigation, Methodology; **R.H.G.:** methodology, writing – original draft; **W.Y.:** validation; **L.M.F.:** writing – review and editing; **Q.Y.F.:** writing – review and editing; **F.J.L.:** validation; **D.R.C.:** writing – review and editing; **F.W.:** formal analysis; **W.Y.P.:** data Curation; **X.Z.C.:** conceptualisation, project administration; **X.H.X.:** supervision; **W.F.:** conceptualisation, funding acquisition. All authors have read and agreed to the published version of the manuscript.

## Ethics Statement

Animal experiments were conducted in accordance with ethical guidelines and approved by the ethics committee of Shanghai University of Traditional Chinese Medicine (Approval NO. PZSHUTCM2402240002).

## Consent

All authors consent to publish this manuscript.

## Conflicts of Interest

The authors declare no conflicts of interest.

## Supporting information


**Figure S1.** Evaluation of the effect of Rg1 on mitochondrial function in OGD/R SH‐SY5Y cells. Cells were subjected to OGD followed by 24 h reperfusion with varying concentrations of Rg1 (4, 8 and 16 μM). (A) Representative flow cytometry images of mtROS detected by MitoSOX Red, with quantification of mtROS levels. (B, C) Representative flow cytometry images and quantification of mitochondrial membrane potential, measured by JC‐1 staining. (D) Changes in cellular ATP production. Data are expressed as mean ± SD. **p < 0.01; ****p < 0.0001 versus DMSO group or indicated group. ^####^ *p* < 0.0001 versus Ctrl group. “NS” indicates no significant difference.
**Figure S2.** Evaluation of the effect of Rg1 on mitochondrial function in OGD/R SK‐N‐AS cells. Cells were subjected to OGD followed by 24 h reperfusion with varying concentrations of Rg1 (4, 8 and 16 μM). (A) Representative flow cytometry images of mtROS detected by MitoSOX Red, with quantification of mtROS levels. (B, C) Representative flow cytometry images and quantification of mitochondrial membrane potential, measured by JC‐1 staining. (D) Changes in cellular ATP production. Data are expressed as mean ± SD. *p < 0.05; **p < 0.01; ***p < 0.001 versus DMSO group or indicated group. ^####^ *p* < 0.0001 versus Ctrl group. “NS” indicates no significant difference.
**Figure S3.** Evaluation of the long‐term effects of Rg1 on cytotoxicity and mitochondrial function in SH‐SY5Y cells subjected to OGD/R. Cells were exposed to OGD followed by 48 h or 96 h of reperfusion with varying concentrations of Rg1 (4, 8 and 16 μM). Cell viability was assessed using the CCK‐8 assay after 48 h (A) and 96 h (B) of reperfusion. Representative flow cytometry images and quantification of mitochondrial membrane potential measured by JC‐1 staining after 48 h (C, E) and 96 h (D, F) of reperfusion. Data are expressed as mean ± SD. **p < 0.01; ***p < 0.001; ****p < 0.0001 versus DMSO group or indicated group. ^####^ *p* < 0.0001 versus Ctrl group.
**Figure S4.** Evaluation of the long‐term effects of Rg1 on cytotoxicity and mitochondrial function in SK‐N‐AS cells subjected to OGD/R. Cells were exposed to OGD followed by 48 h or 96 h of reperfusion with varying concentrations of Rg1 (4, 8 and 16 μM). Cell viability was assessed using the CCK‐8 assay after 48 h (A) and 96 h (B) of reperfusion. Representative flow cytometry images and quantification of mitochondrial membrane potential measured by JC‐1 staining after 48 h (C, E) and 96 h (D, F) of reperfusion. Data are expressed as mean ± SD. *p < 0.05; **p < 0.01; ***p < 0.001; ****p < 0.0001 versus DMSO group or indicated group. ^###^ *p* < 0.001; ^####^ *p* < 0.0001 versus Ctrl group. “NS” indicates no significant difference.

## Data Availability

The data that support the findings of this study are available from the corresponding author upon reasonable request.
