# Supplementary material for: The Neuroprotective Role of Ginsenoside Rg1 Against Cerebral Ischemia–Reperfusion Damage Through Inhibition of Mitophagy via Blocking Mitophagosome‐Lysosome Fusion
Source: Cell Prolif. 2025 Jun 3;59(1):e70071. doi: 10.1111/cpr.70071 (PMC12774623; doi:10.1111/cpr.70071)
Supplement: Supplementary file 1 — Figure S1. Evaluation of the effect of Rg1 on mitochondrial function in OGD/R SH‐SY5Y cells. Cells were subjected to OGD followed by 24 h reperfusion with varying concentrations of Rg1 (4, 8 and 16 μM). (A) Representative flow cytometry images of mtROS detected by MitoSOX Red, with quantification of mtROS levels. (B, C) Representative flow cytometry images and quantification of mitochondrial membrane potential, measured by JC‐1 staining. (D) Changes in cellular ATP production. Data are expressed as mean ± SD. **p < 0.01; ****p < 0.0001 versus DMSO group or indicated group. #### p < 0.0001 versus Ctrl group. “NS” indicates no significant difference. Figure S2. Evaluation of the effect of Rg1 on mitochondrial function in OGD/R SK‐N‐AS cells. Cells were subjected to OGD followed by 24 h reperfusion with varying concentrations of Rg1 (4, 8 and 16 μM). (A) Representative flow cytometry images of mtROS detected by MitoSOX Red, with quantification of mtROS levels. (B, C) Representative flow cytometry images and quantification of mitochondrial membrane potential, measured by JC‐1 staining. (D) Changes in cellular ATP production. Data are expressed as mean ± SD. *p < 0.05; **p < 0.01; ***p < 0.001 versus DMSO group or indicated group. #### p < 0.0001 versus Ctrl group. “NS” indicates no significant difference. Figure S3. Evaluation of the long‐term effects of Rg1 on cytotoxicity and mitochondrial function in SH‐SY5Y cells subjected to OGD/R. Cells were exposed to OGD followed by 48 h or 96 h of reperfusion with varying concentrations of Rg1 (4, 8 and 16 μM). Cell viability was assessed using the CCK‐8 assay after 48 h (A) and 96 h (B) of reperfusion. Representative flow cytometry images and quantification of mitochondrial membrane potential measured by JC‐1 staining after 48 h (C, E) and 96 h (D, F) of reperfusion. Data are expressed as mean ± SD. **p < 0.01; ***p < 0.001; ****p < 0.0001 versus DMSO group or indicated group. #### p < 0.0001 versus Ctrl group. Figure S4. Eval [file CPR-59-e70071-s001.docx]

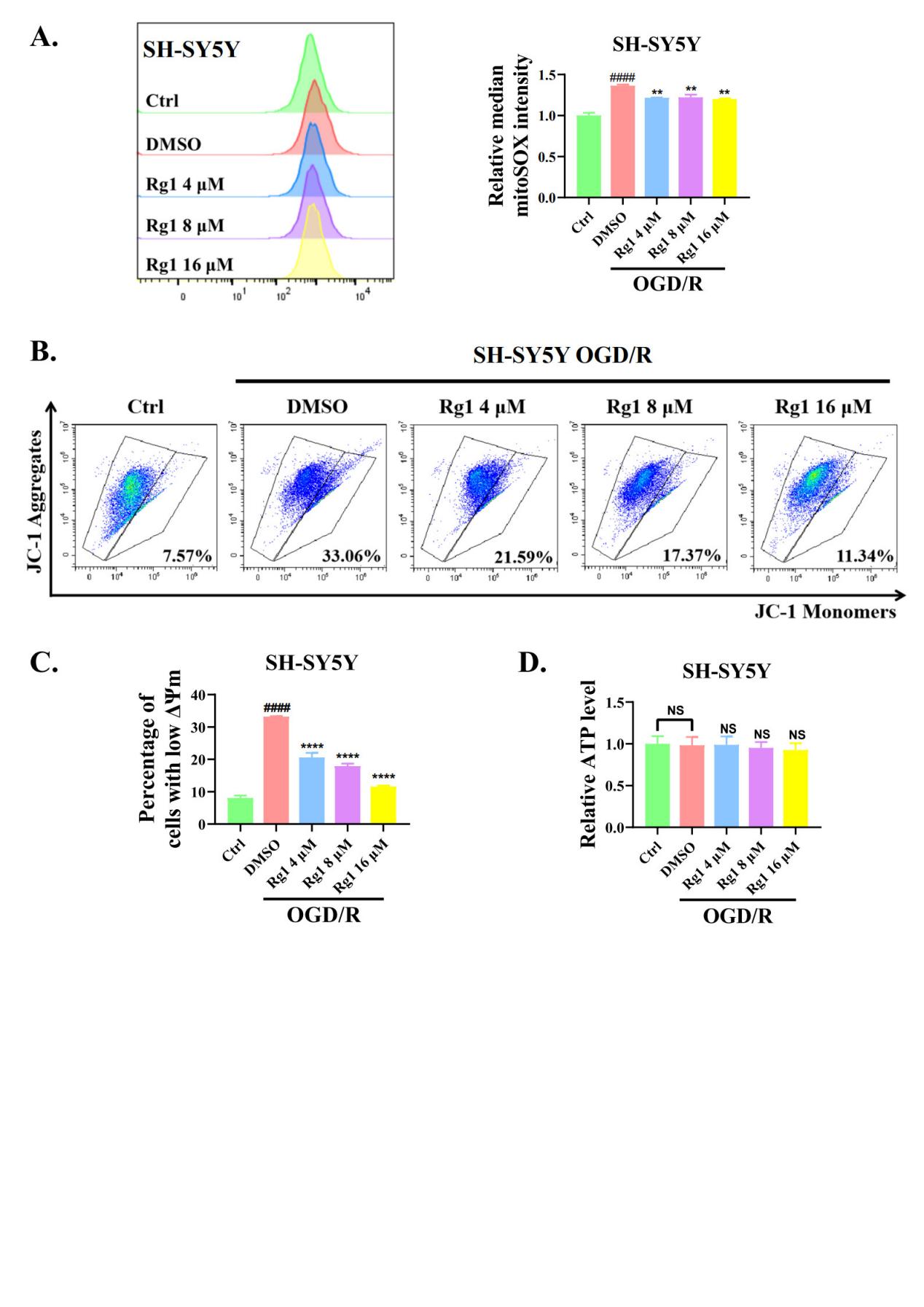


**Figure S1. Evaluation of the effect of Rg1 on mitochondrial function in OGD/R SH-SY5Y cells.** Cells were subjected to OGD followed by 24 h reperfusion with varying concentrations of Rg1 (4, 8 and 16 μM). **A** Representative flow cytometry images of mtROS detected by MitoSOX Red, with quantification of mtROS levels. **B-C** Representative flow cytometry images and quantification of mitochondrial membrane potential, measured by JC-1 staining. **D** Changes in cellular ATP production. Data are expressed as mean ± SD. *******P* < 0.01; *********P* < 0.0001 *versus* DMSO group or indicated group. **^####^***P* < 0.0001 *versus* Ctrl group. "NS" indicates no significant difference.


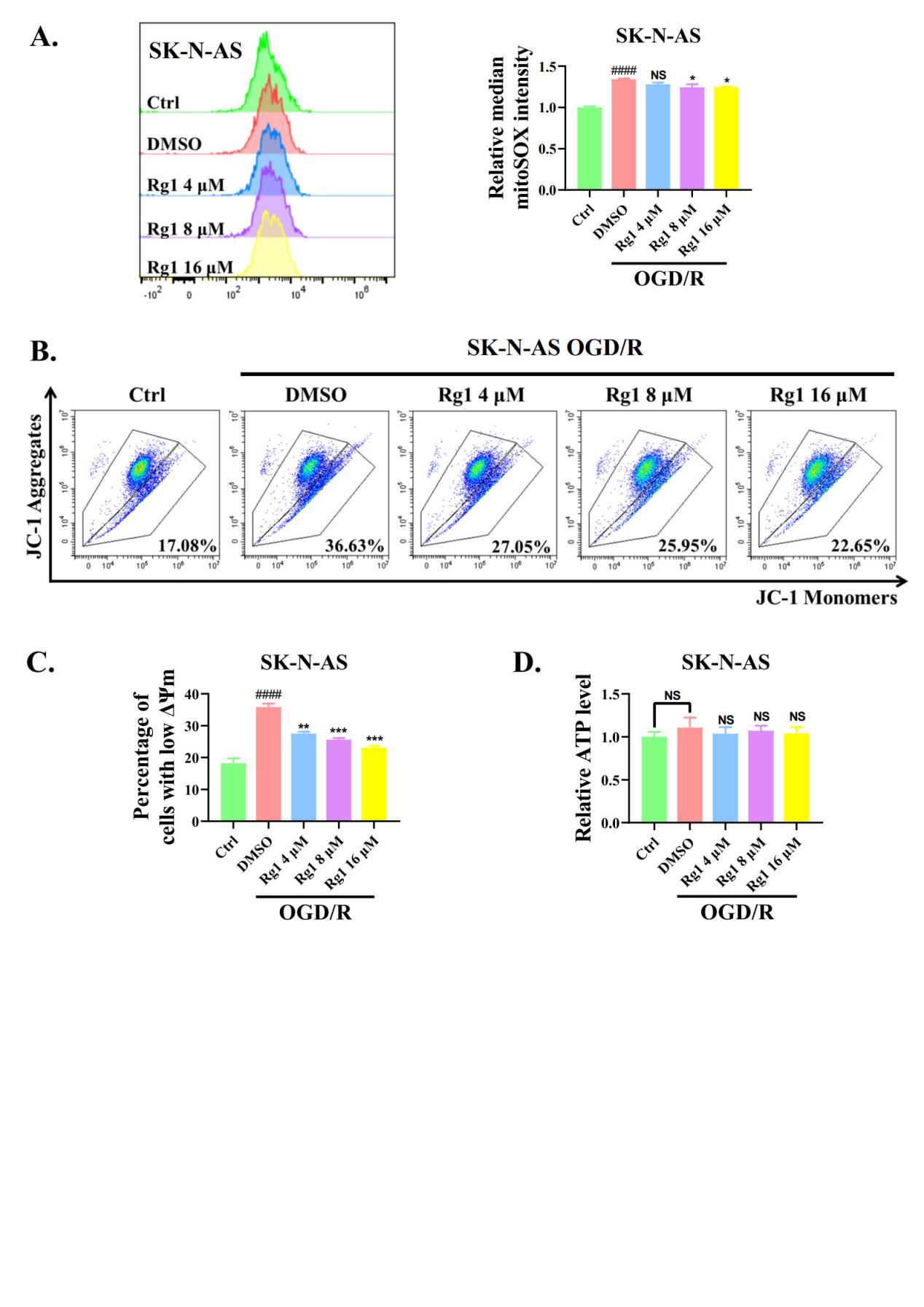


**Figure S2. Evaluation of the effect of Rg1 on mitochondrial function in OGD/R SK-N-AS cells.** Cells were subjected to OGD followed by 24 h reperfusion with varying concentrations of Rg1 (4, 8 and 16 μM). **A** Representative flow cytometry images of mtROS detected by MitoSOX Red, with quantification of mtROS levels. **B-C** Representative flow cytometry images and quantification of mitochondrial membrane potential, measured by JC-1 staining. **D** Changes in cellular ATP production. Data are expressed as mean ± SD. ******P* < 0.05; *******P* < 0.01; ********P* < 0.001 *versus* DMSO group or indicated group. **^####^***P* < 0.0001 *versus* Ctrl group. "NS" indicates no significant difference.


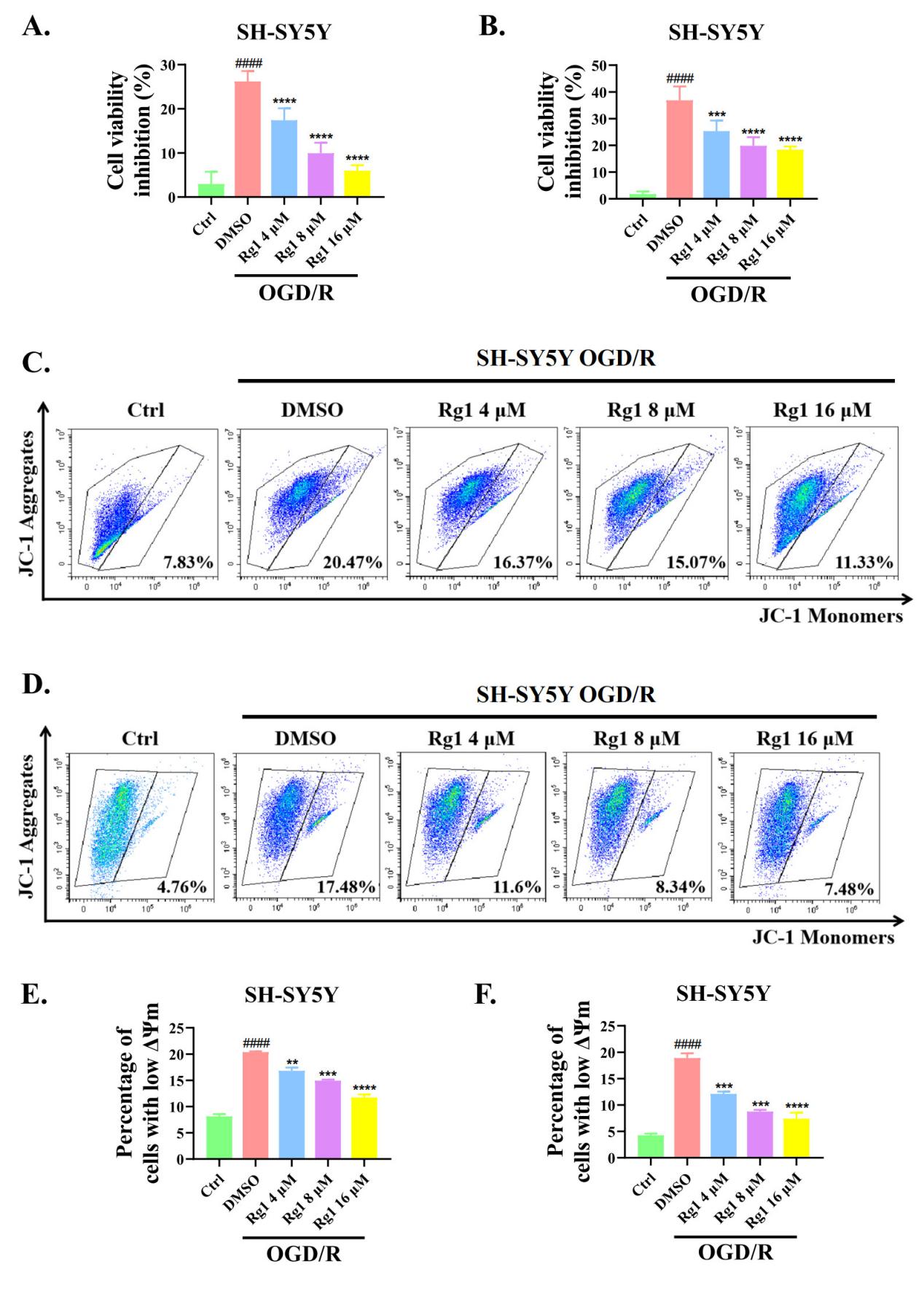


**Figure S3. Evaluation of the long-term effects of Rg1 on cytotoxicity and mitochondrial function in SH-SY5Y cells subjected to OGD/R.** Cells were exposed to OGD followed by 48 h or 96 h of reperfusion with varying concentrations of Rg1 (4, 8 and 16 μM). Cell viability was assessed using the CCK-8 assay after 48 h (**A**) and 96 h (**B**) of reperfusion. Representative flow cytometry images and quantification of mitochondrial membrane potential measured by JC-1 staining after 48 h (**C, E**) and 96 h (**D, F)** of reperfusion. Data are expressed as mean ± SD. *******P* < 0.01; ********P* < 0.001; *********P* < 0.0001 *versus* DMSO group or indicated group. **^####^***P* < 0.0001 *versus* Ctrl group.


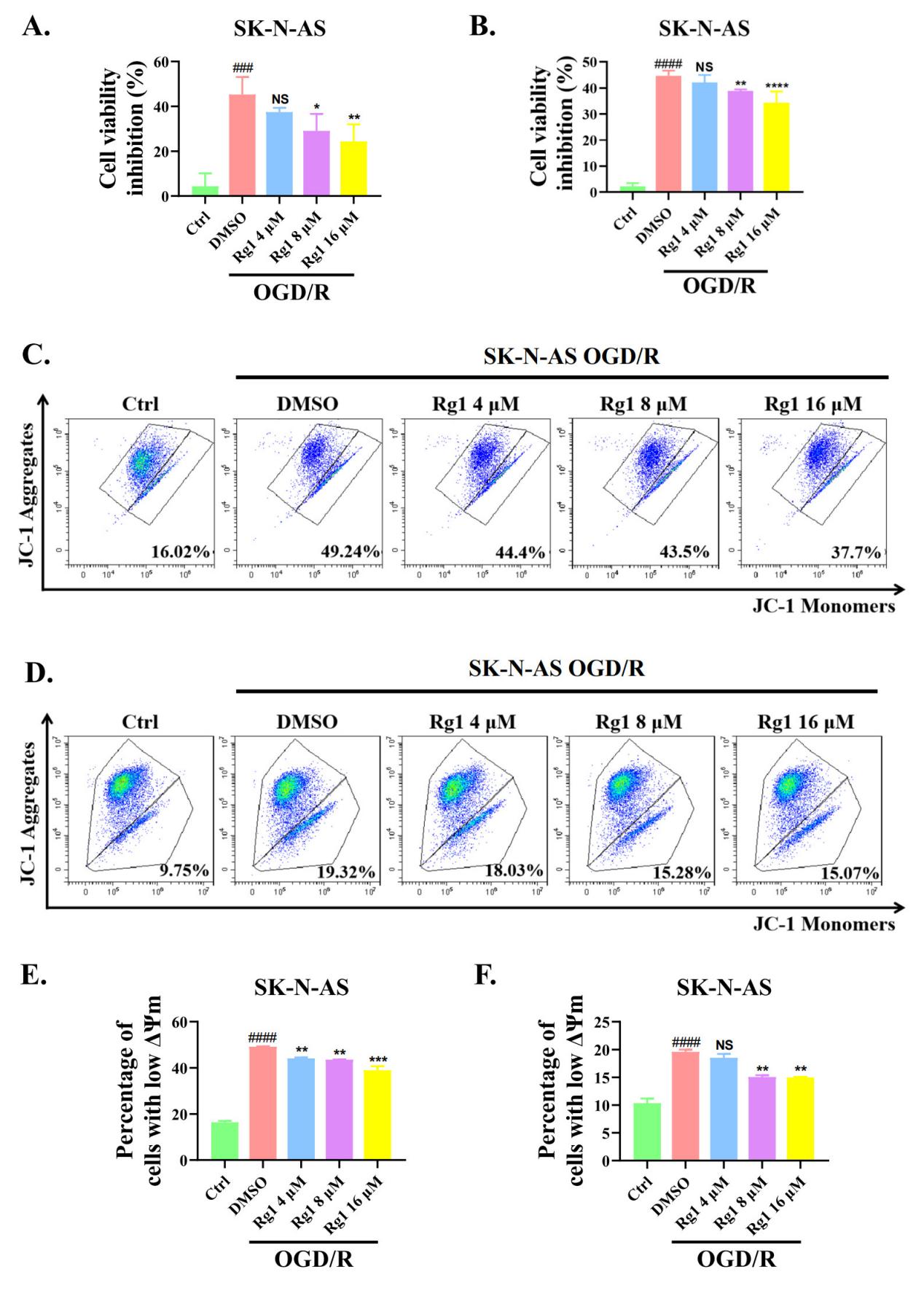


**Figure S4. Evaluation of the long-term effects of Rg1 on cytotoxicity and mitochondrial function in SK-N-AS cells subjected to OGD/R.** Cells were exposed to OGD followed by 48 h or 96 h of reperfusion with varying concentrations of Rg1 (4, 8 and 16 μM). Cell viability was assessed using the CCK-8 assay after 48 h (**A**) and 96 h (**B**) of reperfusion. Representative flow cytometry images and quantification of mitochondrial membrane potential measured by JC-1 staining after 48 h (**C, E**) and 96 h (**D, F)** of reperfusion. Data are expressed as mean ± SD. ******P* < 0.05; *******P* < 0.01; ********P* < 0.001; *********P* < 0.0001 *versus* DMSO group or indicated group. **^###^***P* < 0.001; **^####^***P* < 0.0001 *versus* Ctrl group. "NS" indicates no significant difference.
